# Catecholamine Surge in the Emergency Department: A Tech-Knowledgy-Preventable Pheochromocytoma Crisis

**DOI:** 10.1016/j.mcpdig.2023.01.002

**Published:** 2023-02-18

**Authors:** Samantha Pabich, Peter Kleinschmidt, Joseph Halfpap, Jamie Hess

**Affiliations:** a Department of Medicine, University of Wisconsin School of Medicine and Public Health, Madison, WI; b Department of Emergency Medicine, University of Wisconsin School of Medicine and Public Health, Madison, WI

## Abstract

A patient with a known pheochromocytoma was admitted to the emergency department with hypertension and headache. Because of the disease rarity, the potential medication–disease interactions were not recognized. The patient received a dose of prochlorperazine to treat their headache. Prochlorperazine is a potential trigger of pheochromocytoma crisis. The patient developed worsening hypertension, pulmonary edema, and demand non-ST segment elevation myocardial infarction.

In response, our health system has created a best-practice advisory within our electronic medical records that can alert a provider to interactions between medications and pheochromocytoma. We are looking to expand this practice to include other pathologies with dangerous medication interactions, and we recommend other health systems create similar initiatives.

Pheochromocytoma is such a rare disease that average physicians might need to practice for 125 years to have a reasonable chance of encountering it in their career^1^. Rare diseases may remain undiagnosed by the majority of medical providers. Modal decision support in electronic health records (pop-up alerts) can facilitate the recognition of these risks when providers are not familiar with rare conditions. A 2017 systematic review demonstrated that drug–condition alerts can reduce inappropriate prescription of medications in common conditions (i.e. advanced age, chronic kidney disease)^2^. However, few studies report on how alerts can be used effectively for rare conditions.

## Case Presentation

A patient developed exercise-induced headaches with emesis in their early 40s. As their condition progressed 3 years later, the patient discovered a National Institutes of Health case series on this phenomenon in patients with pheochromocytoma and requested to be tested for this rare condition. Serum metanephrine was 386 pg/mL (7 times the normal upper limit), and serum normetanephrine was 3009 pg/mL (20 times the normal upper limit). Computed tomography demonstrated a 4.9 × 4.4 × 4.3 cm mass in the left adrenal gland of 33 Hounsfield-units radiodensity, with heterogeneous hyperenhancement and washout. The patient was diagnosed with pheochromocytoma and referred to the endocrinology department. At the first visit, the patient was educated about the pathophysiology of the condition and prescribed Prazosin 1 mg twice daily to blockade alpha receptors and prevent hypertension from the intermittent catecholamine surges that characterize the disease^3^. Although they were advised to inform their endocrinologist on starting any new medications, they were not provided with an explicit list of medications to avoid. Surgical referral was initiated to plan for adrenalectomy.

The patient exhibited a symptomatic catecholamine surge with symptoms 4 days later at home. This was not unexpected as they were not fully alpha blocked by this point. They contacted the on-call endocrinologist and reported that their heart rate was 122 beats per minute (BPM) and blood pressure was 152/99 mmHg. However, they reported headache and visual changes. The patient was referred to the emergency department (ED).

The endocrinologist informed the ED physician that the patient was newly diagnosed with pheochromocytoma and recommended ongoing contact with the endocrine service for management. On arrival, the patient was examined by a different ED physician. The blood pressure on arrival (156/105 mmHg) was similar to that at home and to several other blood pressures that had been documented in the months that preceded the diagnosis. Triage notes documented that the team was aware of the pheochromocytoma and that the patient had ongoing headache. Shortly thereafter, intravenous prochlorperazine was prescribed to treat the headache. The patient was hesitant to receive this medication and considered refusing it because of the rarity and complexity of pheochromocytoma. They were administered the medication after being informed that it would improve symptoms. Within minutes, the patient’s systolic blood pressure increased from 156 mmHg to 230 mmHg, and heart rate accelerated from 105 BPM to 165 BPM. Shortly thereafter, they developed respiratory distress, with the work-up demonstrating pulmonary edema and demand non-ST segment elevation myocardial infarction. They required admission to the intensive care unit and bilevel-positive airway pressure support.

The precise temporal correlation with the administration of the medication and the increase in heart rate and blood pressure exhibited that the patient had a pheochromocytoma crisis triggered by prochlorperazine. The patient recovered fully without any long-standing adverse effects. They were managed with alpha blockade and eventually beta blockade, and underwent adrenalectomy approximately 1 month later.

## Discussion

Although the emergency medicine team was aware of the patient’s pheochromocytoma diagnosis, they were not aware of the danger posed to this pathology by an otherwise innocuous ubiquitous medication. Although drug–drug and drug–allergy interaction alerts are common in most modern electronic health record systems, disease-specific alerts might not be considered. If a patient exhibited a diagnosis of “Pheochromocytoma” on their problem list, the electronic medical record might have created a warning if a provider had attempted to order beta blockers, dopaminergics, sympathomimetics, opioids, tricyclics, selective serotonin reuptake inhibitors, monoamine oxidase inhibitors, corticosteroids, glucagon, or neuromuscular blockade^1^. Similarly, a chart entry “Migraine Aura” could generate a warning if an oral contraceptive was ordered, and an entry of “Renal Artery Stenosis, bilateral” could suggest against the use of an angiotensin-converting enzyme-inhibitor or angiotensin receptor blocker.

Our institution has now taken steps to add a pop-up warning if a contraindicated medication class is ordered for a patient with an active diagnosis of pheochromocytoma (Figure 1). Of the 8,068,658 orders entered in inpatient and outpatient encounters during the first 12 months after implementation, this alert was activated 137 times on 24 patients with past or current pheochromocytomas. In 65/137 cases, the medication was either directly discontinued within the alert, or the alert was canceled to then modify orders. In 62/137 cases, the alert was overridden. However, the next steps in these cases were not determined. In 16 of these overrides, the reason given for inappropriately displaying the alert was that the diagnosis remained active on the problem list after the patients had undergone adrenalectomy.

**Figure:**
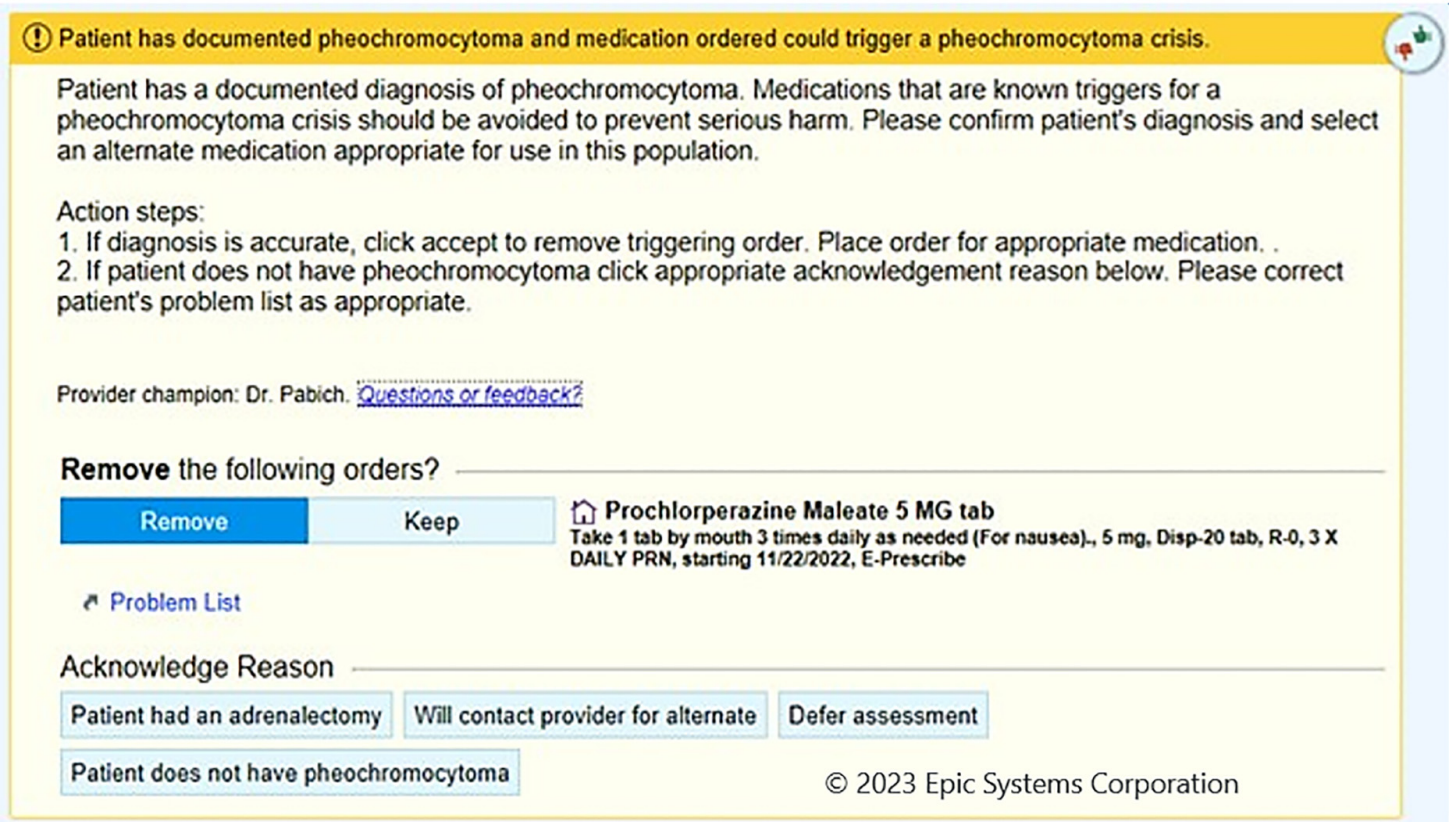
Image of the Best-Practice Advisory pop-up that now appears in our institution’s electronic medical record when a medication that can cause pheochromocytoma crisis is ordered for a patient with a documented pheochromocytoma.

Although organizations must be judicious about the use of alerts, appropriate warnings tailored to high-risk situations may alleviate the risk of adverse drug events. The often cited “5 rights of clinical decision support” (the right information, to the right person, in the right intervention format, through the right channel, at the right time in workflow)^4^ can guide successful implementation of these types of alerts. This case illustrates a unique example where an uncommon disease could be given greater attention by a carefully designed alert.

## Potential Competing Interests

The authors report no competing interests.

## Ethics Statement

The patient was presented with the University of Wisconsin Office of Compliance Authorization for Disclosure of Medical Information for Publication or Conference Presentation form, and gave written approval for dissemination of their case presentation. In order to preserve anonymity, this form will not be disseminated.

Our information was determined not to represent Human Subjects Research by the University of Wisconsin IRB Form HRP 310 Human Subjects Research Determination because the data collected did not pertain to individual patients, but rather to the functionality of a provider alert tool. Assessment of the function of this tool is a part of ongoing quality improvement in our institution, and is not conducted for research purposes.
